# Toxicity of benzodiazepines in the treatment of insomnia disorders in older adults: a systematic literature review

**DOI:** 10.3325/cmj.2024.65.146

**Published:** 2024-04

**Authors:** Annemarie Kim Kozole Smid, Ajda Mlakar, Vita Štukovnik

**Affiliations:** 1 PhD student at the Department of Psychology, University of Maribor, Faculty of Arts, Maribor, Slovenia; 2 University of Ljubljana, Faculty of Medicine, Department of Public Health, Ljubljana, Slovenia; 3 Department of Psychology, University of Maribor, Faculty of Arts, Maribor, Slovenia

## Abstract

**Aim:**

To review the literature data on the prevalence of benzodiazepines abuse and poisoning in older adults; the prevalence of polypharmacy with benzodiazepines in this demographic; and determine whether benzodiazepine anxiolytics or hypnotics were more frequently implicated in the cases of abuse and poisoning.

**Methods:**

We searched PubMed and Scopus for relevant studies published from January 1, 2013, to May 1, 2023. Twelve studies were included in the final selection.

**Results:**

The review highlights the diverse prevalence rates of benzodiazepine abuse and poisoning in the older adult population. Benzodiazepine anxiolytics were more frequently associated with negative outcomes than benzodiazepine hypnotics. Concurrent use of benzodiazepines, benzodiazepine-related medications, and opioids was reported, although these medications were not the only ones commonly used by the elderly.

**Conclusion:**

It is essential to increase awareness about adhering to prescribed pharmacological therapies to mitigate issues related to drug abuse and poisoning among older adults.

Insomnia is a substantial public health concern (1). The International Classification of Sleep Disorders, third edition (ICSD-3), defines insomnia as a persistent difficulty with falling asleep, maintaining sleep, or reduced quality of sleep, occurring despite adequate opportunities and conditions for sleep. It is a recognized risk factor for impaired daily functioning, substance abuse, depression, other psychiatric disorders, chronic pain, and various other health issues, including obesity, high blood pressure, cardiovascular diseases, and dementia (2). Effective treatment of insomnia is particularly vital in older adults, where age-related changes in sleep, such as circadian rhythm dysregulation and changes in sleep architecture, can aggravate the condition (3).

Benzodiazepines are among several medications for insomnia treatment approved by the United States Food and Drug Administration (4). Only short-term pharmacotherapy for insomnia is recommended, typically lasting four to five weeks (5,6). Prolonged use of medications can lead to dependence. Additionally, using medications in doses inconsistent with prescribed therapy can also lead to dependence (7).

Benzodiazepines are safe and effective when prescribed and used judiciously (8). Nonetheless, in prescribing these medications for older adults with insomnia, it is crucial to consider age-related pharmacokinetic changes, like altered drug metabolism (9) and pharmacodynamic shifts. For example, alterations in the GABA neurotransmitter system lead to increased sensitivity to adverse effects, including ataxia, sedation, and cognitive impairments (10). In this regard, potential issues may arise concerning medication abuse and poisoning among older adults, involving adverse effects, and, in severe cases, leading to death (11). This is particularly relevant as the elderly often concurrently use multiple medications (12).

The clinical manifestations of benzodiazepine poisoning are often more severe in older individuals, frequently leading to coma and increased incidence of complications and longer hospital stays (13). More cases of respiratory failure were reported in older patients; however, there was no significant difference between the groups of older and younger patients. These outcomes may be attributed to age-related pharmacokinetic changes or heightened sensitivity due to compromised organ function, comorbidities, and drug interactions.

The aim of this study is to systematically review the existing literature on the prevalence of benzodiazepine abuse and poisoning in older adult population with insomnia. We aimed to obtain data on the prevalence of benzodiazepines abuse and poisoning in older adults; the prevalence of polypharmacy with benzodiazepines in this demographic; and investigate whether abuse and poisoning occurred more frequently with benzodiazepine hypnotics or benzodiazepine anxiolytics.

## Methods

Preferred Reporting Items for Systematic Reviews and Meta-Analyses (PRISMA) was used as a guideline for writing and reporting. Publication scanning was conducted for a specified time period through an electronic search of the PubMed and Scopus databases.

### Study design

We performed a brief systematic literature review concentrating on instances of benzodiazepine poisoning and abuse among older adults diagnosed with insomnia. Published literature generally defines late adulthood as the period from 65 years onwards. Our systematic review included studies involving individuals aged 65 and above, with one exception that included individuals aged 61 and above (7,14-24).

### Inclusion criteria

Studies were selected based on the following inclusion criteria:

1. Study sample: older adults, aged 61 and above.

2. Health condition: insomnia treatment.

3. Medication: treatment for insomnia with benzodiazepines, either solely or in combination with other insomnia medications.

4. Study types: controlled clinical studies, randomized controlled trials, descriptive and cross-sectional studies, cohort studies, and case studies.

5. Time period: studies conducted in the last 10 years (2013-2023).

6. Outcome: instances of benzodiazepine abuse and poisoning

7. Language: Slovenian or English.

### Databases and search string

We searched the PubMed and Scopus databases for relevant literature published from January 1, 2013, to May 1, 2023. A uniform search string was employed in both databases: “benzodiazepine and (elderly or 'older adult*' or senior*) and insomnia and (overdose or misuse or abuse or intoxication or poisoning)”.

### Search strategy

**Figure 1:**
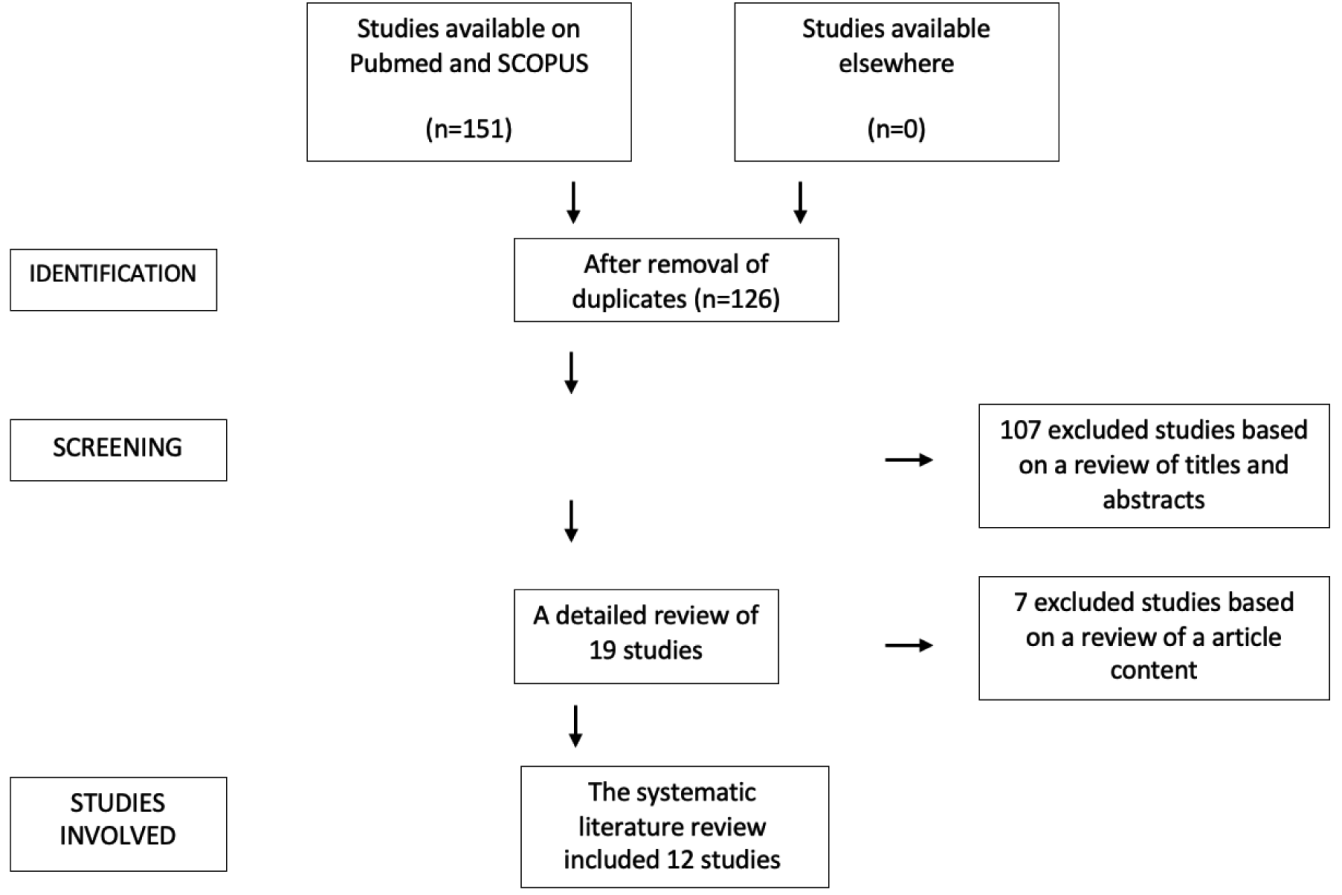
The selection process of studies included in the systematic literature review.

The publication selection involved a three-step process (Figure 1). Initially, 151 articles were identified (126 after removing duplicates) using a predefined search string across PubMed and Scopus databases. After title screening, 51 articles were excluded. In the second step, we examined the abstracts of the remaining articles and, excluded further 56 articles. The full texts of the remaining 19 articles were assessed, resulting in the inclusion of 12 articles (Table 1).

**Table 1:** Studies included in the systematic literature review

Research	Age group	Number of Participants	Concurrent use of different benzodiazepines or other medications	Benzodiazepines	Type of benzodiazepines	Adverse outcome	Treatment for insomnia	Main findings
**Schepis 2021 (** **22** **)**	65+ years	223 520 participants (14 885 participants older than 65 years)	/	Asked about the use of benzodiazepines, benzodiazepine-related drugs, or barbiturates.	/	Drug abuse, dependence	It is assumed that a lot of participants have insomnia, as most of them report using sedatives or hypnotics for better sleep.	Motives for misuse of prescribed sedatives varied by age group, with the highest rates of motives for self-treatment (ie, sleep and/or relaxation) among those older than 65 years (82.7%). 17.3% of seniors older than 65 years reported misuse of medications for other purposes.
**El Zahran 2022 (** **19** **)**	66+ years	244 participants (67 participants aged 66+ years)	Most patients reported the presence of chronic diseases such as hypertension, coronary artery disease, cancer, thyroid disease, benign prostatic hyperplasia, migraine, diabetes, different lung diseases, arrhythmias, autoimmune diseases, epilepsy, dyslipidaemia, kidney disease, fibromyalgia, etc.	Alprazolam, bromazepam, clonazepam, lorazepam, diazepam, chlordiazepoxide	Anxiolytics	Drug abuse, inappropriate use of medications	45.5% of participants reported using benzodiazepines for treatment of insomnia; however, 32 of 47.8% of participants aged 66+ years reported benzodiazepine use disorder.	47.8% of seniors older than 66 years reported via validated questionnaire misuse or inappropriate use of medications.
**Niznik 2023 (** **21** **)**	65-69 years, 70-79 years, 80-89 years, 90+ years	25 141 participants older than 65 years	13.4% of individuals with chronic benzodiazepine use also concurrently used opioids, and 7.9% used more medications. The concurrent use of benzodiazepines and opioids due to the presence of concurrent medical conditions, including chronic pain, anxiety, and insomnia.	Alprazolam, clonazepam, lorazepam.	Anxiolytics	Drug abuse	26.72% of participants reported using benzodiazepines for treatment of insomnia.	Chronic use of benzodiazepines was reported in 29.6% of seniors aged from 65 to 69 years, in 47.2% of those aged from 70 to 79 years, in 20.5% of those aged from 80 to 89 years, and in 2.6% of those older than 90 years.
**Cook 2018 (** **24** **)**	65+ years	11 663 participants (18+ years)	/	/	/	Drug abuse, dependence	On average, 12% of participants experienced sleep disturbances. Sleep disturbances were significantly represented among individuals abusing or improperly using benzodiazepines and among those at risk of benzodiazepine dependence.	There was significantly more likely to abuse or improper use of benzodiazepines among seniors older than 65 years.
**Sakshaug 2017 (** **14** **)**	65+ years	28 884 participants older than 65 years	Concurrent use of benzodiazepines and benzodiazepine-related drugs, as well as opioids.	Diazepam and oxazepam (as anxiolytics), nitrazepam and flunitrazepam (as hypnotics).	Most of the participants used anxiolytics, with a smaller percentage of them using hypnotics.	Drug abuse, inappropriate use of medications.	Possible presence of treatment of insomnia (as some individuals used hypnotics).	Concurrent use of benzodiazepines and benzodiazepine-related drugs is the lowest for people older than 65 years compared with other age groups (18-39 years and 40-64 years). Concurrent use of benzodiazepines and other related drugs –between 1.5% (females) and 1.8% (males) in the population of participants older than 65 years. Concurrent use of benzodiazepines increased with more intensive treatment with benzodiazepine-related drugs. Concurrent use of these drugs with benzodiazepines and opioids can cause additional negative effects on the central nervous system and can increase the risk of different side effects.
**Yamamoto 2021 (** **23** **)**	65-90 years	707 participants	On average, participants simultaneously used two different medications. On average 1.4 people in the study group using hypnotics and 1.3 people in the study group using anxiolytics. Participants did not use a combination of hypnotics, benzodiazepine-related drugs, and anxiolytics.	Brotizolam, clonazepam, cloxazolam, estazolam, etizolam, flunitrazepam, flurazepam, haloxazolam, loflazepate, lormetazepam, nimetazepam, nitrazepam, quazepam, rilmazafon, triazolam, diazepam, alprazolam, bromazepam, chlordiazepoxide.	Hypnotics and anxiolytics.	Drug abuse – not following the instructions and other problematic use of medications.	Patients are treated in a sleep disorders clinic.	The study confirmed a statistically significant non-compliance with the usage regimen and problematic use of benzodiazepine hypnotics among the elderly (aged 65-90 years).
**Ray 2021 (** **15** **)**	65+ years	400 924 participants	No simultaneous use of multiple benzodiazepines, but simultaneous use of benzodiazepines and opioids.	Alprazolam, clonazepam, estazolam, flurazepam, lorazepam, quazepam, temazepam, triazolam.	Hypnotics and anxiolytics.	Mortality	Elderly with sleep disorders or insomnia also included.	Concurrent use of benzodiazepines and opioids and benzodiazepine-related drugs in seniors older than 65 years was associated with increased outpatient and overall mortality. Total mortality increased more than two times, even after excluding deaths from overdose, which indicates that simultaneous exposure to benzodiazepines and opioids poses a significant health risk to the elderly.
**Díaz-Gutiérrez 2018 (** **20** **)**	Older adults (exact age not specified)	654 participants older than 65 years	On average, patients used 5.6 different medications. 44 participants took 7 or more medications simultaneously. Most of the participants (N = 118) took one type of benzodiazepine or benzodiazepine-related drugs, some took two (N = 19), or three (N = 3).	Lormetazepam, lorazepam, alprazolam, diazepam, clorazepate, ketazolam, brotizolam	Hypnotics and anxiolytics.	Falls associated with abuse of benzodiazepines.	Lormetazepam was most commonly prescribed, which is a hypnotic intended for treating insomnia. Zolpidem is also a hypnotic for treating insomnia but is not a benzodiazepine.	40.6% of older adults consumed a higher dose than defined or recommended daily dose. Of the 57 patients using either benzodiazepines or benzodiazepine-related drugs in doses higher than recommended, 53 experienced trauma, and 33 required hospitalization.
**Yen 2015 (** **7** **)**	65+ years	139 participants	Concurrent use of different medications was not allowed	Estazolam and flunitrazepam.	Hypnotics.	Inappropriate use of hypnotics and dependence.	Participants used hypnotics for insomnia treatment. 9.4% of participants had a diagnosis of sleep disorder.	28.8% of participants reported addiction, and 7.9% reported inappropriate use of hypnotics.
**Cremaschi 2019 (** **16** **)**	70 years (male)	1 participant	Concurrent use of benzodiazepines and benzodiazepine-related drugs.	Alprazolam and zolpidem (a benzodiazepine-related drug).	Anxiolytic.	Drug abuse, excessive use (overdose).	He used benzodiazepines for treatment of insomnia.	A case study of benzodiazepine poisoning. He was taken to the emergency department due to poisoning. Without timely intervention, he could have died.
**Tahiri 2017 (** **18** **)**	61+ years	780 participants (237 participants older than 61 years)	/	/	/	Drug abuse, inappropriate drug use.	113 (42.6%) participants had a diagnosis of insomnia.	58.6% of seniors older than 61 years reported abuse or inappropriate use of benzodiazepines.
**Kay 2016 (** **17** **)**	From 44 to 87 years (M = 66 years); average age of individuals with a suicide attempt was M = 63 years	135 participants (72 with a previous suicide attempt, 28 with a plan of suicide, and 35 individuals without a plan or previous suicide attempt)	/	27 individuals out of 72 who attempted suicide had consumed benzodiazepines but the suicide attempt could not be explained by the consumption of benzodiazepines.	/	Suicide attempt.	Individuals who attempted suicide suffered from severe insomnia compared to individuals with suicidal ideations or individuals without a suicide attempt.	The suicide attempt could not be explained by the consumption of benzodiazepines.

### Coding

Data were gathered on the name of the first author, publication year, specific age groups of the older adults involved, concurrent use of different benzodiazepine types or other medications, specific benzodiazepines investigated, categorization of hypnotics vs anxiolytics, adverse drug effects examined, confirmation of insomnia treatment, and key findings (Table 1).

Our coding framework encompassed four primary categories derived from the included studies: sociodemographic data (age), diagnosis/treatment of insomnia, pharmacological treatment (involving benzodiazepines or benzodiazepines with other medications), and adverse outcomes associated with these medications.

## Results

### Review of literature

Out of 126 hits remaining after removing duplicates, we examined publications at two levels (review of titles and abstracts). Using the designated search string, we thoroughly reviewed the content of 19 studies. Among these, three were excluded due to unsuitable sample characteristics, and one was excluded for being a literature review. Despite potential relevance, two studies were further excluded due to a certain level of deviation from our research concept of interest. Consequently, 12 studies were included in our final systematic literature review.

### Main findings

*Intoxications with benzodiazepines in the elderly.* Potential poisoning may occur as a result of simultaneous use of multiple medications. Ray et al (15) observed that the simultaneous use of benzodiazepines with opioids and benzodiazepine-related medications in individuals aged 65 and above correlated with increased outpatient and overall mortality. Even after excluding deaths due to overdose, the simultaneous use still resulted in more than a 2-fold increase in overall mortality among the elderly, signifying a health risk. The potential for benzodiazepine poisoning was also highlighted in the case study by Cremaschi et al (16), which reported on a 70-year-old man who attempted suicide by overdosing on benzodiazepines and ended up in a coma. Kay et al (17) also noted a link between benzodiazepine use and suicide attempts, although they indicated that suicide attempts could not be solely attributed to benzodiazepine use.

*Abuse of benzodiazepines among the elderly.* Benzodiazepine abuse rates among the elderly were as high as 58.6% in those over 61 (19). El Zahran et al (19) reported a slightly lower rate of 47.8% for abuse or inappropriate medication use among the elderly aged 66 and above. Similarly, Díaz-Gutiérrez et al (20) found that 40.6% of older adults consumed a daily dose that was higher than the defined or recommended, and Niznik et al (21) reported 47.2% chronic use of benzodiazepines in individuals aged 70 to 79. In the latter study, the rate of benzodiazepine abuse in the age groups of 65-69 years, 80-89 years, and 90+ years was 29.6%, 20.5%, and 2.6%, respectively. On the other hand, Schepis et al (22) documented a slightly lower use of sedatives for purposes other than treatment, namely 17.3%, in individuals aged over 65. In the study by Yen et al (7), 28.8% of participants aged over 65 reported dependence, and 7.9% reported inappropriate use of benzodiazepine hypnotics. Similarly, Yamamoto et al (23) observed pronounced non-compliance with the regimen and problematic use of benzodiazepine hypnotics among the elderly aged 65 to 90. Cook et al (24) reported a significant likelihood of abuse or inappropriate use of benzodiazepines in individuals aged over 65.

### Secondary findings

*Simultaneous use of medications.* Studies primarily reported on the concurrent use of benzodiazepines, benzodiazepine-related medications, and opioids (14-16,20,21). Studies reporting on the average intake of medications in the elderly indicated, for example, the simultaneous use of two different medications (23) or an average of 5.6 different medications (20). The mentioned studies revealed that the majority of participants were taking one type of benzodiazepine or benzodiazepine-related medication, while some were taking two or even three. Most of the participants in the study by El Zahran et al (19) reported various comorbidities (eg, hypertension, coronary artery disease, cancer, thyroid disease, benign prostatic hyperplasia, migraines, diabetes, lung diseases, arrhythmias, autoimmune diseases, epilepsy, dyslipidemia, kidney diseases, fibromyalgia, and other illnesses), necessitating the use of multiple concurrent medications.

*Anxiolytics and hypnotics*. Although the findings were somewhat inconsistent, misuse and poisoning were slightly more strongly associated with the use of anxiolytics (14,16,19-21). Most studies reported that abuse and poisoning were associated with the use of anxiolytics, particularly alprazolam, diazepam, lorazepam, and clonazepam. The use of other anxiolytics like bromazepam, chlordiazepoxide, oxazepam, clorazepate, and ketazolam was also noted. Concerning hypnotics, the studies reported that the abuse and poisoning were associated with the use of hypnotics such as brotizolam, cloxazolam, estazolam, etizolam, flunitrazepam, flurazepam, haloxazolam, loflazepate, lormetazepam, nimetazepam, nitrazepam, quazepam, rilmazafone, triazolam, and temazepam.

## Discussion

In this study, the rates for inappropriate use of benzodiazepines ranged from relatively low for hypnotics in those over 65 (7) to over 50% in aged over 61 (18). The lowest abuse rate was recorded by Niznik et al (21) in the age group of 90 years and older. This result could be a consequence of the generally low representation of this age group (3.21% of the total sample, with only 25 individuals chronically using benzodiazepines). A relatively high rate of benzodiazepine abuse (58.6%) was recorded by Tahiri et al (18) in the elderly from Kosovo. Researchers from the neighboring country of Albania recorded 76.4% of inappropriate use of these drugs using the same measurement instruments (25). A substantial prevalence was also observed in the elderly aged over 66 in Lebanon (19). Additionally, almost the same result for the age group of 70-79 years was recorded in North Carolina, United States (21), where the population certainly has access to information about the consequences of improper use but may face an issue of increased medication prescription. Namely, the total number of medication prescriptions in the US in 2021 amounted to 6474 million, compared with 3953 million in 2009 (26). On the other hand, interestingly, Schepis et al (22) reported a lower abuse of tranquilizers (17.3%) across the US population for purposes other than prescribed medications in individuals aged over 65. An even lower level of abuse (7.9%) was recorded in Taiwan (7), but there was a higher rate of dependence on hypnotics. The reason for this could be the low number of participants. Yamamoto et al (23) and Cook et al (24) also reported a considerable problematic or improper use of benzodiazepines in the elderly aged over 65. Age-related changes in sleep contribute to the onset of insomnia in the elderly and specifically the use of benzodiazepines. However, the reason for improper use may also lie in coping with everyday concerns, challenges, and changes that come with age. Many older individuals face a narrowing of their social circle and thus feelings of loneliness (27), grief (28), and other physical and psychological changes.

Regarding the secondary outcome of the study, Díaz-Gutiérrez et al (20) reported an average consumption of 5.6 different medications. Yamamoto et al (23) noted a simultaneous consumption of an average of two different medications in the elderly aged between 65 and 90 years. Sakshaug et al (14) also reported a relatively low rate of concurrent use of benzodiazepines and related medications in those over 65 (1.8% for men and 1.5% for women). Notably, most studies reported the simultaneous use of benzodiazepines, benzodiazepine-related medications, and opioids (14-16,20,21), though these are not the only medications commonly used by the elderly (eg, medications for blood pressure, cholesterol, diabetes, etc). Ray et al (15) emphasized the increased non-hospital and overall mortality associated with the simultaneous use of benzodiazepines, benzodiazepine-related medications, and opioids in those over 65, suggesting potential toxicity, especially given the possible subclinical deterioration of liver and kidney function in the elderly (13). A case study by Cremaschi et al (16) reported on a suicide attempt by benzodiazepines poisoning, possibly due to the age-related changes and a lack of emotional support at the end of life. The elderly may be more determined in their suicide attempts than younger individuals (13).

Although the findings were somewhat inconsistent, misuse and poisoning were slightly more strongly associated with the use of anxiolytics (14,16,19-21). The latter, in general, represent a group of medications that is more frequently abused (29). Geulayov et al (30) noted the higher toxicity of temazepam, a benzodiazepine hypnotic, and zopiclone or zolpidem compared with diazepam, a benzodiazepine anxiolytic. Regardless of the classification, vigilant monitoring is essential to ensure that the elderly receive appropriate pharmacological therapy.

### Limitations and potential for further research

A potential limitation of this review is the reliance on only two databases. Incorporating additional databases could yield more studies and provide a more comprehensive view of the topic. Another limitation could also be the disproportionate focus on the studies that investigated abuse or inappropriate use of benzodiazepines, as opposed to poisoning or ingestion of excessive doses of benzodiazepines and potential mortality in the elderly. Further research could involve reviewing the remaining databases and investigating the poisoning or ingestion of excessive doses among the elderly. In the future, more research attention could be dedicated to poisoning resulting from the use of benzodiazepines in conjunction with other common medications frequently taken by the elderly (eg, medications for blood pressure, cholesterol, diabetes, etc.).

### Conclusion

The findings of this brief systematic literature review underscore a varying prevalence - from a few percent to over fifty percent - and potential concerns regarding abuse or inappropriate use of benzodiazepine in general, as well as poisoning among older adults. The association of benzodiazepine anxiolytics with benzodiazepine abuse and poisoning was slightly more pronounced compared with that of benzodiazepine hypnotics. These insights underscore the importance of raising awareness about benzodiazepine-related issues in the older adult population and the critical need for adherence to prescribed pharmacological therapies for benzodiazepine use in general.
